# Next Generation Sequencing-Based Profiling of Cell Free DNA in Patients with Advanced Non-Small Cell Lung Cancer: Advantages and Pitfalls

**DOI:** 10.3390/cancers12123804

**Published:** 2020-12-17

**Authors:** Riziero Esposito Abate, Daniela Frezzetti, Monica Rosaria Maiello, Marianna Gallo, Rosa Camerlingo, Antonella De Luca, Rossella De Cecio, Alessandro Morabito, Nicola Normanno

**Affiliations:** 1 Cell Biology and Biotherapy Unit, Istituto Nazionale Tumori-IRCCS-Fondazione G. Pascale, 80131 Naples, Italy; r.espositoabate@istitutotumori.na.it (R.E.A.); d.frezzetti@istitutotumori.na.it (D.F.); m.maiello@istitutotumori.na.it (M.R.M.); marianna.gallo@istitutotumori.na.it (M.G.); r.camerlingo@istitutotumori.na.it (R.C.); a.deluca@istitutotumori.na.it (A.D.L.); 2 Department of Pathology, Istituto Nazionale Tumori-IRCCS-Fondazione G. Pascale, 80131 Naples, Italy; r.dececio@istitutotumori.na.it; 3 Department of Thoracic Medical Oncology, Istituto Nazionale Tumori-IRCCS-Fondazione G. Pascale, 80131 Naples, Italy; a.morabito@istitutotumori.na.it

**Keywords:** next generation sequencing, cfDNA, NSCLC, targeted therapy, liquid biopsy

## Abstract

**Simple Summary:**

Genomic profiling of non-small cell lung cancer (NSCLC) patients offers the possibility of therapeutic intervention with target-based agents. The analysis of circulating free DNA (cfDNA) through next generation sequencing (NGS) technologies is emerging as a powerful tool to assess the whole tumor molecular landscape of NSCLC patients and characterize spatial and temporal tumor heterogeneity. Moreover, cfDNA NGS testing allows the monitoring of therapy response and the early identification of resistance mechanisms. In this review, we describe the importance of implementation of cfDNA NGS testing in routine clinical practice to improve targeted diagnostic procedures and personalized therapies in NSCLC patients.

**Abstract:**

Lung cancer (LC) is the main cause of death for cancer worldwide and non-small cell lung cancer (NSCLC) represents the most common histology. The discovery of genomic alterations in driver genes that offer the possibility of therapeutic intervention has completely changed the approach to the diagnosis and therapy of advanced NSCLC patients, and tumor molecular profiling has become mandatory for the choice of the most appropriate therapeutic strategy. However, in approximately 30% of NSCLC patients tumor tissue is inadequate for biomarker analysis. The development of highly sensitive next generation sequencing (NGS) technologies for the analysis of circulating cell-free DNA (cfDNA) is emerging as a valuable alternative to assess tumor molecular landscape in case of tissue unavailability. Additionally, cfDNA NGS testing can better recapitulate NSCLC heterogeneity as compared with tissue testing. In this review we describe the main advantages and limits of using NGS-based cfDNA analysis to guide the therapeutic decision-making process in advanced NSCLC patients, to monitor the response to therapy and to identify mechanisms of resistance early. Therefore, we provide evidence that the implementation of cfDNA NGS testing in clinical research and in the clinical practice can significantly improve precision medicine approaches in patients with advanced NSCLC.

## 1. Introduction

In the last years, the identification of driver genomic alterations that represent targets for therapeutic intervention has revolutionized the management of non-small cell lung cancer (NSCLC) patients. The assessment of the mutational status of predictive biomarkers, such as Epidermal Growth Factor Receptor (*EGFR*), Anaplastic Lymphoma Kinase (*ALK*), ROS Proto-Oncogene 1 Receptor Tyrosine Kinase (*ROS1*), and V-Raf Murine Sarcoma Viral Oncogene Homolog B (*BRAF*), plays a crucial role in the identification of the most suitable therapeutic strategy for NSCLC patients in the current clinical practice. Kinase inhibitors have been developed for targeting tumors that carry mutations in these biomarkers and they have significantly improved the survival of NSCLC patients [1,2,3,4,5,6,7,8,9]. However, the spectrum of genomic biomarkers and matched targeted therapies for NSCLC patients is rapidly expanding. Novel agents demonstrated significant clinical activity in tumors carrying either *MET* exon 14 mutations [10,11], *RET* fusions [12], and *NTRK* rearrangements [13,14], and have been already approved by the Food and Drug Administration (FDA). Interesting preliminary data are also available on the activity of drugs targeting the KRAS G12C variant [15] and *ERBB2* mutations in lung cancer patients [16].

In routine clinical practice, tumor tissue biopsy represents the gold standard for biomarker testing. Molecular profiling of tumor tissue samples takes advantage of highly standardized methodologies. However, tumor tissue samples can result inadequate for biomarker analysis because the DNA extracted is insufficient and/or degraded. Moreover, in approximately 30% of NSCLC patients, tumor tissue sampling is unfeasible either at diagnosis or at disease progression for different reasons, including risk of complications, tumor inaccessibility, and patient refusal [17].

To overcome these limitations, researchers have explored the possibility to use liquid biopsy as an alternative method for biomarker testing. In particular, the circulating cell-free DNA (cfDNA) isolated from different matrices, such as peripheral blood, sputum, and pleural fluid, contains variable levels of tumor-derived DNA that is defined circulating tumor DNA (ctDNA) [18,19]. Apoptosis and necrosis have been proposed as the main mechanisms of DNA release by tumor cells into body fluids, due to the specific nuclease-dependent fragmentation pattern characteristic of ctDNA (160 bp) [20]. In plasma samples, ctDNA is diluted in the normal DNA released in the peripheral blood by dividing cells, such as skin, gastrointestinal, and hematological cells [21,22]. In some cases, the ctDNA is a very small fraction of the total cfDNA, making the use of highly sensitive techniques necessary for its detection.

In NSCLC patients, ctDNA levels strongly correlate with several clinical and pathological features, including tumor volume, histology, and the extent of necrosis [20,23]. A correlation between the site of the disease and the levels of ctDNA has also been demonstrated in metastatic NSCLC. In particular, patients with intrathoracic disease only have usually lower levels of ctDNA as compared with patients with extrathoracic disease [24,25].

Liquid biopsy testing has several advantages compared to traditional diagnostic techniques; since it is a minimally invasive procedure, it can be repeated to follow tumor evolution over time and, most importantly, it is able to better recapitulate tumor heterogeneity (Table 1). In fact, tumors are constituted by different clones, which grow with different proliferation rates under the pressure of the tumor microenvironment and of administered targeted therapies [26]. While the analysis of tissue sample offers a snapshot of the tumor only in the place where it is biopsied, cfDNA testing allows to characterize the complete and real-time molecular profile of the tumor [26,27]. Moreover, through repeated blood sampling, it allows the early detection of molecular mechanisms of resistance to targeted therapies [28].

Several studies demonstrated the suitability of the assessment of molecular alterations by cfDNA analysis in patients with advanced NSCLC (aNSCLC), leading the European Medicine Agency to approve cfDNA testing for *EGFR* mutations analysis in case of tumor tissue unavailability [29]. *EGFR* mutations in cfDNA are tested in many laboratories by using either Real-Time PCR or emulsion PCR techniques, including digital droplet PCR (ddPCR) and Bead Emulsion Amplification and Magnetic (BEAMing) [22]. These techniques have a high sensitivity, which makes them ideal for the identification of mutations at very low allelic frequencies (AFs), up to 0.01%. However, the main limit of these technologies is that they can interrogate few loci per analysis. Therefore, they are not suitable to assess the multiple and complex genomic alterations that are emerging as relevant targets for precision medicine in NSCLC. In contrast, massive parallel sequencing, better known as next generation sequencing (NGS), has the advantage to allow sequencing dozens to hundreds of genes per test. For this reason, NGS-based technologies are emerging as the best approach to determine the genomic profile of NSCLC patients starting from cfDNA. A list of the main advantages of NGS-based cfDNA testing compared to tumor tissue biopsy is shown in Table 1.

In this review article, we summarize the current knowledge on the development and clinical application of cfDNA NGS testing in the management of aNSCLC patients.

## 2. Next Generation Sequencing Technologies for cfDNA Testing

NGS is a broad term that refers to different approaches for sequencing from few genes up to the whole genome. Indeed, whole genome sequencing (WGS) and whole exome sequencing (WES) are able to detect genetic alterations in the entire genome and in the exon regions, respectively (Table 2). Several researchers are trying to apply these techniques to cfDNA analysis, but the high costs, the long turnaround times, and the requirement of qualified bioinformatics experts for the analysis of the large quantity of data generated from WGS and WES, limit their large-scale use (Table 2). For these reasons, WGS and WES are being explored in the field of advanced research and they are not yet applicable in the clinical setting and on large cohorts of patients for clinical research purposes [30,31,32,33]. On the other hand, targeted sequencing of defined regions of the genome is able to identify somatic genetic alterations with a high level of sensitivity and a low number of false negatives. Targeted sequencing is based on two main different strategies for library preparation, the amplicon-based and capture-based technologies (Table 2). In the amplicon-based methods, target genes are first amplified using specific primers binding to regions of interest and then they are marked with unique sequences called barcodes. In the capture-based technologies, DNA is previously fragmented, ligated to unique adapters, and then regions of interest are selected by specific probes [34]. Among the amplicon-based techniques used for the analysis of cfDNA, the TAgged-Amplicon deep Sequencing (TAm-Seq) technology employs special tagged primers to amplify regions of interest. To avoid errors and allelic loss, primers bind to target regions in the first step of amplification to ensure the correct amplification of the genes of interest, and then samples are amplified individually [35]. This technology can reveal genetic alterations at 2% of mutation AF (MAF), with a sensitivity over 97% [36]. Recently, this technology has been upgraded to enhanced TAm-Seq (eTAm-Seq), which has an improved sensitivity (0.25% of MAF) and it is able to detect also complex genetic alterations, such as copy number variations (CNVs) and indels [37]. Another amplicon-based approach is the Safe-Sequencing System (Safe-SeqS), which takes advantage of primers with a unique identifier. This technology reduces the error rate of at least 70-fold with a sensitivity of 98% for detecting tumor mutations [38]. Finally, the AmpliSeq HD technique can detect single nucleotide variations (SNVs), indels and gene fusions with a limit of detection up to 0.1%. This method consists of an amplification step using a single pool of oligonucleotide primer pairs, with each pair designed to amplify a specified genomic region. The error rate is very low thanks to the unique molecular tags linked to the gene-specific primers, that allow to eliminate the errors generated during the library preparation and the sequencing process [39,40] (Table 2). The capture-based CAncer Personalized Profiling by deep Sequencing (CAPP-Seq) is based on hybridization capture with biotinylated DNA oligonucleotides, called “selectors”, targeting specific regions of interest mutated in over 95% of tumors. This method is able to detect genetic alterations at a very low frequency (0.02%) [41]. However, its error rate is estimated in 50% of sequenced genomic positions, due to sequencing artifacts and low cfDNA input [42]. This limit has been overcome by the introduction of integrated Digital Error Suppression (iDES), which exploits molecular barcodes tagging double-stranded DNA molecules with unique identifiers. In this manner, iDES technology reaches a sensitivity of 92% and a specificity of 96% on plasma samples [42] (Table 2).

## 3. NGS-Based cfDNA Analysis for Guiding Precision Medicine in NSCLC

The use of NGS technologies applied to cfDNA testing for NSCLC patients is continuously expanding and improves the detection of actionable genomic alterations compared to the analysis of tumor tissue alone, thus allowing the improvement of personalized therapy with target-based agents. Here we describe the main studies demonstrating the suitability of NGS-based cfDNA analysis and the main advantages of its use in clinical routine procedures.

### 3.1. Genomic Profiling of NSCLC by cfDNA Analysis

Several studies demonstrated that NGS testing of plasma provides a genomic profile of NSCLC patients similar to tissue testing and, even more importantly, that the integration of plasma NGS assay into routine management increases the detection of clinically relevant mutations [24,43,44].

Most data on genomic profiling of NSCLC patients with cfDNA NGS testing have been generated with the Guardant360^®^ panel (Guardant Health). This panel is able to assess genetic alterations (SNVs, indels, CNV, fusions) in 73 cancer related genes [45,46]. The results of cfDNA analysis with the Guardant360^®^ panel of over 8000 plasma samples from NSCLC patients have been recently published [43]. Genetic alterations were identified in 86% of samples, with 57.2% showing more than one mutation. In some cases, multiple *EGFR* and *KRAS* mutations were detected simultaneously [43]. Furthermore, the study demonstrated that driver mutations in 18 known lung-associated oncogenes, such as *BRAF*, *RET*, *ALK,* and *ROS1*, follow a mutual exclusivity pattern. Differently, a small group of patients had simultaneously driver mutations in *EGFR* and *KRAS* genes. Interestingly, these patients had significantly more additional nonstandard mutations compared to patients with mutations of *EGFR* or *KRAS* alone [43]. Importantly, in a subset of 1288 cases for which both tissue and cfDNA testing results were known, the testing of cfDNA increased by 65% the detection rate of actionable genomic alterations (Table 3). Such increase was mainly due to the fact that in a large fraction of cases the tumor was not genotyped or was under-genotyped due to insufficient tissue [43]. Similar results were previously reported by Leighl and coworkers, who found that, using cfDNA analysis in addition to tumor tissue testing, the detection of actionable genomic alterations increased by 48% in a cohort of 282 aNSCLC patients [44] (Table 3). An increase from 20.5% of cases with actionable genomic alterations detected in tumor tissue alone to 35.8% following the addition of plasma testing has also been reported in another independent study in NSCLC patients [24] (Table 3). Interestingly, the frequency of actionable genomic alterations was higher in a cohort of plasma-only tested patients as compared with tissue-tested cases (33% versus 20.5%). Taken together, these findings suggest that cfDNA testing can replace or integrate tumor tissue testing, when the amount of tissue available for testing is limited.

A significant advantage in the use of NGS over methods to detect single biomarkers is the possibility to identify co-occurring mutations that might limit the activity of targeted agents [47]. For example, genomic analysis with a 68-gene NGS panel of cfDNA from 1122 *EGFR* mutant stage III/IV NSCLC patients before EGFR Tyrosine Kinase Inhibitors (TKI) therapy revealed in 92.9% of the cases the presence of co-occurring variants, of which 89.8% had verified or likely functional effects based on in silico prediction. Interestingly, the mean number of additional variants detected in cfDNA was lower in patients responding to subsequent EGFR TKI treatments compared to nonresponders, suggesting that such alterations could be linked to intrinsic resistance to anti-EGFR therapy [48]. Similarly, Jin and colleagues performed NGS analysis of 416 cancer relevant genes from 69 untreated *EGFR* mutant advanced lung adenocarcinoma patients, both on tumor tissue DNA and on cfDNA from plasma or pleural effusions. Patients with shorter progression-free survival (PFS) (<6 months) carried known variants (i.e., *PIK3CA* and *NRAS* mutations, *AKT1* and *HGF* amplifications) and novel mutations potentially associated to intrinsic resistance, such as missense mutations (*CDC73* and *SMAD4*), frameshift indels (i.e., *RB1*, *DNMT3A*, *STK11*, and *ATR*), gene fusions (*CDKN2B-PATA31D1* and *NFKBIA-OR11H12*), and copy number gains (i.e., *CCNE1* and *MCL1*) [49]. Again, in a retrospective cohort study, targeted NGS analysis of cfDNA obtained from 58 metastatic NSCLC patients before first-line treatment with EGFR TKI demonstrated that the presence of concomitant genetic alterations were significantly associated with shorter PFS and overall survival (OS) [50].

### 3.2. Outcome of Patients Receiving Targeted Therapy Matched to cfDNA Genomic Profiling

The outcome of NSCLC patients treated with targeted agents based on the genomic profile of the cfDNA is similar to the results observed in patients receiving therapy on the basis of tumor tissue testing. In a cohort of 88 patients tested on cfDNA with the Guardant360^®^ panel, 72.3% of the 25 aNSCLC patients who received a targeted therapy matched to the cfDNA genotype achieved stable disease ≥6 months or partial response [51]. In this study, NSCLC patients with at least one alteration detected in the cfDNA and a MAF ≥5% showed a shorter OS than patients with MAF <5% (median 4.2 months vs. not reached value up to 7.5 months), confirming a prognostic role of ctDNA levels [51]. A 86% disease control rate (DCR) at 3 months and a median PFS of 14.8 months was observed in a retrospective analysis of 81 aNSCLC patients treated with targeted agents, based on tagged amplicon-based NGS testing of cfDNA [52]. In particular, the 3-months DCR was 87% for *EGFR* mutant patients, 100% for *ALK/ROS1* fusion positive, and 50% for BRAF V600E mutant patients [52]. Similarly, 36/42 (85.7%) aNSCLC patients who received a targeted therapy matched to the plasma genotype, assessed with the Guardant360^®^ panel, achieved a complete or a partial response or stable disease [24]. Importantly, no correlation was found between the activity of targeted therapy and the MAF in the cfDNA, since also patients with low MAF achieved a relevant clinical response [24,52].

The above-summarized data derive mainly from retrospective analyses or single-center experiences. However, results from prospective clinical trials further support the use of NGS-based cfDNA testing for therapeutic purposes. In the phase 2 clinical trial of tepotinib in aNSCLC patients carrying *MET* exon 14 skipping mutations, prospective testing was performed on cfDNA with the Guardant360^®^ panel or on tumor tissue with the NGS Oncomine Focus Assay [11]. Notably, the response rate was similar in the two groups: 48% in patients tested on liquid biopsy and 50% in tumor tissue tested group. In addition, median PFS was 8.5 months in the group analyzed with liquid biopsy and 11.0 months in the group with mutations detected on tumor tissue biopsy [11].

### 3.3. Factors Affecting the Concordance between Tumor Tissue and cfDNA Testing

Despite the above-summarized findings strongly support the use of NGS for cfDNA testing, some concerns on this approach derive from conflicting results that have been reported on the concordance between tumor tissue and cfDNA testing. High concordance rates ranging from 80% to almost 100% have been observed for the main actionable genomic alterations, such as *EGFR, ALK, MET, ROS1, RET, ERBB2,* and *BRAF* variants in several studies [24,44]. Moreover, the concordance was significantly higher in patients at diagnosis than in patients at progression, being 88.9% and 70.2%, respectively [24]. In contrast, a 60–70% overall concordance rate has been reported in studies in which all the genetic alterations identified in tumor specimens and plasma samples were considered, including *TP53* and *KRAS* mutations [39,40,51,53]. Importantly, the overall concordance rates were higher when the time elapsed between tissue and plasma sampling was short (62.5% vs. 43.3%; cut-off 1.3 months) [51].

**Figure 1:**
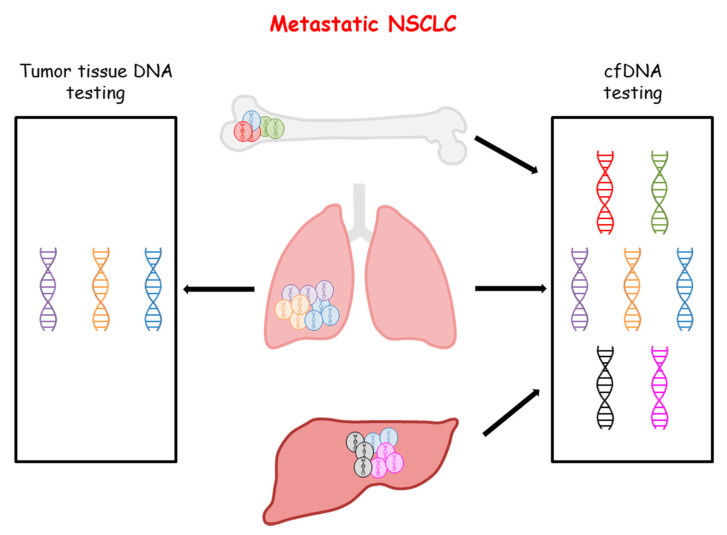
Representation of tumor heterogeneity detected with tumor tissue and cfDNA testing for metastatic NSCLC patients.

Discrepancies between genetic profiles deriving from tumor tissue and cfDNA testing may be due to a number of factors. A fraction of NSCLC does not shed or sheds small amounts of DNA, which might be below the threshold of sensitivity of current techniques. Indeed, a fraction of nonshedder patients has been identified in almost all studies on cfDNA testing of LC patients [54,55]. Lack of vascularization, low proliferation rates, and anatomical barriers are likely to play a role in this phenomenon. In addition, clonal hematopoietic mutations of indeterminate potential (CHIP) might also significantly affect the concordance between tissue and plasma testing. In particular, mutations in *KRAS* or *TP53* genes might be associated with CHIP and are difficult to distinguish from cancer-associated mutations [56]. The frequency of detection of CHIP is correlated with the age of the patients and the sensitivity of testing methods [57]. Furthermore, NGS profiling of cfDNA from healthy donors revealed that the presence of *TP53* mutations did not correlate with subsequent tumor insurgence after a follow up of 10 years [58]. Sequencing artifacts have also been reported when cfDNA is analyzed with NGS-based technologies [59]. Finally, tumor heterogeneity is likely to play a relevant role in the discordance between plasma and tissue testing [40]. In fact, tumor heterogeneity has been demonstrated to occur in different tumor types including NSCLC [60]. This phenomenon might lead to different genomic patterns within the same tumor lesion or among different metastatic sites. Therefore, we might expect that cfDNA testing will better depict such heterogeneity in patients with metastatic disease as compared with the testing of a small fragment of a single tumor site (Figure 1). In this respect, testing both tumor tissue and cfDNA might provide complementary information on tumor heterogeneity.

## 4. Use of NGS-Based cfDNA Testing to Track the Acquired Resistance to Targeted Therapies in Advanced NSCLC

The use of NGS technologies for cfDNA testing finds a challenging field of application in the identification of molecular alterations responsible for the development of resistance to targeted treatments [61]. All NSCLC patients who initially respond to targeted therapy develop eventually resistance to treatment. The identification of the molecular mechanisms driving resistance might offer the possibility of therapeutic intervention thanks to the availability of novel drugs that can overcome resistance.

In the following paragraphs, we describe the application of NGS-based analysis of liquid biopsy for detecting and monitoring the resistance to targeted therapies commonly used in the management of aNSCLC patients.

### 4.1. Monitoring Resistance to EGFR Tyrosine Kinase Inhibitors

First or second generation EGFR TKIs, including gefitinib, erlotinib, and afatinib, have for a long time represented the recommended treatment for patients carrying *EGFR* activating mutations, although are now being replaced in first-line by third-generation EGFR TKIs, such as osimertinib [62]. The majority of *EGFR* mutant patients initially responding to EGFR TKIs develop resistance within 10–12 months of treatment [63,64]. In this respect, the most frequent mechanism of acquired resistance to first and second generation EGFR TKIs consists of the acquisition of the EGFR T790M mutation (50–60% of cases). Its detection after progression became clinically relevant with the availability of osimertinib, that showed significant clinical activity in T790M-positive patients [65]. Actually, the identification of the EGFR T790M mutation through cfDNA testing has entered into routine clinical practice in aNSCLC patients progressing upon first-line EGFR TKIs treatment [66]. Importantly, spatially heterogeneous expression of the T790M mutation has been demonstrated in some patients progressing on TKI treatment [67]. In these cases, cfDNA testing can provide complementary information on T790M status to tissue biopsy [68].

NGS coupled with mutation enrichment PCR have proven to identify the EGFR T790M mutation in liquid biopsies of NSCLC patients with high sensitivity (93% for plasma and urine samples of the recommended volume of 90–100 mL) and specificity (94% and 96% for plasma and urine, respectively) [69]. Remon and colleagues prospectively assessed the efficacy of osimertinib in patients progressing on EGFR TKIs treatment by T790M detection in cfDNA with eTAm-Seq [70]. Response rate (62.5%) and 12-months PFS (52%) for T790M cfDNA positive patients following osimertinib was comparable to that reported for patients treated on the basis of T790M detection in tumor biopsy [70]. In a meta-analysis of 1639 aNSCLC patients from 21 studies, the pooled sensitivity and specificity of NGS-based ctDNA T790M testing resulted, respectively, of 0.87 (95% CI 0.76–0.95) and of 0.89 (95% CI 0.82–0.94), higher than other detection methods (Real-Time PCR, ddPCR), suggesting an higher performance of NGS [71]. More recently, a tag-based NGS panel was used to identify T790M in plasma from NSCLC patients, resulting in a significantly improved detection rate of NGS compared to Real-Time PCR (42.85% versus 21.4%, respectively). Moreover, NGS was able to detect mutations at very low AFs (up to 0.07%), with a reduction of the amount of false negative cases requiring repeated tumor biopsy and a decrease of turnaround times as compared to other technologies [72].

The main advantage of using NGS in this setting is its ability to detect co-occurring genomic alterations that could drive resistance to targeted therapies. In fact, acquired resistance to targeted therapy is often multiclonal. NGS testing of cfDNA revealed that, in approximately 50% of T790M-positive cases, additional genomic alterations can be identified in genes that are frequently associated with resistance to EGFR TKIs [73]. Interestingly, the analysis of the cfDNA from T790M-positive patients with targeted sequencing showed a higher frequency of co-occurring genomic alterations in osimertinib nonresponder patients as compared with osimertinib responders, with particular regard to cell cycle-related genes [48].

NGS testing of cfDNA has been also fundamental to identify the mechanisms of resistance to novel third generation EGFR TKIs. For example, this approach allowed to discover the EGFR C797S mutation in NSCLC patients with acquired resistance to osimertinib [74].

Acquired resistance of NSCLC patients to EGFR TKIs treatment is exerted not only through EGFR-dependent mechanisms, but also through molecular alterations in other genes, such as *MET* and *ERBB2* amplifications, *PI3KCA*, *BRAF*, and *KRAS* mutations, and through phenotypic change, such as small cell lung cancer (SCLC) transformation [75,76]. In this regard, Guibert and colleagues demonstrated the ability of amplicon-based NGS to identify both genomic alterations commonly associated to EGFR TKI resistance (i.e., *MET* and *ERBB2* amplifications) and novel mutations (i.e., EGFR Q791P) in serial plasma samples from osimertinib treated NSCLC patients, even several months before clinical progression [77]. In another study, 77 NSCLC patients treated with the third generation EGFR TKI rociletinib were subjected to cfDNA NGS testing before treatment and at progression. Again, the use of a 70-gene NGS panel allowed the identification of multiple and novel resistance alterations, evidencing a significant heterogeneity of gene mutations and of variant types both before and after the acquisition of resistance [78]. In a more recent report comparing ctDNA analysis using conventional methods (Real-Time PCR and ddPCR) and targeted NGS, the successful ability of a 11-gene NGS panel to identify therapeutic targets and monitor drug resistance in two independent cohorts of aNSCLC patients has been demonstrated, with turnaround times of only four days and a workflow for data interpretation easily implementable in clinical routine procedures [79].

Interestingly, recent evidence suggests that NGS-based liquid biopsy might be helpful in the characterization of patients with transformation into SCLC in response to anti-EGFR therapy, even though tissue rebiopsy is required to confirm the change at the histological level. Actually, NGS testing of plasma samples from NSCLC patients progressing following EGFR TKIs treatment allowed the identification of marked copy number changes in cfDNA even months before the confirmation of SCLC transformation, including copy number gain of *MYCL1*, *SOX2,* and *SOX4*. Moreover, loss or mutations of *TP53* and *RB1* genes were also observed [80,81]. Although further studies are required to confirm these observations, the identification of global copy number changes in liquid biopsies might early indicate resistance development in EGFR TKI treated NSCLC patients, indicating the requirement for tissue rebiopsy to eventually confirm SCLC transformation.

### 4.2. Monitoring Resistance to ALK and ROS1 Tyrosine Kinase Inhibitors

NSCLC patients carrying *ALK* or *ROS1* gene fusions benefit from treatment with selective TKIs. Unfortunately, as for anti-EGFR therapy, some patients do not respond to TKIs treatment and eventually all of them progress within 1–3 years of therapy. For *ALK* fusion positive patients progressing on crizotinib, the second generation TKIs ceritinib and brigatinib have been approved [82,83]. Among the principal mechanisms of resistance of *ALK* or *ROS1* positive NSCLC tumors, there are both variants in *ALK* or *ROS1* genes (mutations or copy number gains) and *ALK/ROS1* independent resistance alterations, including *cKIT* amplification and *KRAS* mutations [84,85]. However, a comprehensive characterization of the mechanisms of intrinsic and acquired resistance to ALK and ROS1 inhibitors is still missing.

Compared to EGFR TKIs, fewer studies explored the use of NGS-based liquid biopsy for the detection of resistance mechanisms to ALK and ROS1 inhibitors. Recently, performing a hybrid capture-based NGS assay on plasma of 18 *ROS1* positive NSCLC patients relapsing after crizotinib, Dagogo-Jack and colleagues identified SNVs possibly associated with resistance in 44% of cases (five patients with ROS1 G2032R, one with ROS1 L2026M, one with PIK3CA E545K, and one with BRAF V600E) [86]. More recently, NGS analysis of serial liquid biopsies of an *ALK* rearranged patient upon progression on crizotinib and ceritinib treatment, identified two mutations potentially associated to resistance to these drugs, ALK G1269A and G1202R, respectively [87]. These findings highlight the importance of surveilling tumor clonal evolution during TKIs treatment by cfDNA NGS profiling of *ALK* fusion positive NSCLC patients. Finally, in a large prospective cohort study involving *ALK* and *ROS1* positive NSCLC patients treated with first to third generation TKIs, a targeted amplicon-based NGS assay revealed the presence of cfDNA alterations potentially correlated with resistance, both in *ALK* or *ROS1* genes and in other genes (i.e., *KRAS*, *PI3KCA,* and *PTEN*). Moreover, the absence of cfDNA mutations at TKIs failure was associated with better outcomes, suggesting the potential role of cfDNA testing as a surrogate biomarker for TKI treatment efficacy in *ALK/ROS*1 positive NSCLC patients [88].

### 4.3. Monitoring Resistance to Immune Checkpoint Inhibitors

The introduction of immune-checkpoint inhibitors (ICIs) represented a major breakthrough for the treatment of metastatic NSCLC, with particular regard to Programmed Death 1 (PD-1)/PD-L1 axis inhibitors. However, mechanisms of primary and acquired resistance largely limit the activity of these drugs, with a very low fraction of patients experiencing a prolonged response [89]. In this respect, several mechanisms of resistance to ICIs treatment have been identified and, among those associated with genetic variants of tumor cells, there are alterations of *HLA*, *β2-microglobulin* and *LKB1* genes, *c-Myc* copy number gain, *KRAS* mutations, and deletions of chromosomal regions with consequent neoantigens loss [90].

The availability of NGS-based techniques for the analysis of cfDNA can significantly expand its field of application to monitoring the response to immunotherapy. In this respect, different studies have demonstrated that early changes in the levels of ctDNA are associated with response to ICIs [91,92]. In particular, tracking ctDNA levels in the blood of cancer patients treated with ICIs could be highly relevant to distinguish a true progression from pseudo-progression, an initial increase of tumor size due to immune infiltration as a consequence of ICIs treatment, followed by subsequent response to immunotherapy [93]. Indeed, this possible application has been demonstrated by tracking *KRAS* mutations in cfDNA by ddPCR [94]. The advantage of NGS is that by using large panels, this approach could be extended to a much larger fraction of patients in which a “tracer” mutation could be identified to monitor response to ICIs treatment.

In addition, liquid biopsy testing could provide information on other relevant biomarkers. For example, the evaluation of tumor mutational burden (TMB) in plasma from NSCLC patients has emerged as a potential instrument for the detection of response/resistance to ICIs treatment [95]. In this regard, Gandara and collaborators demonstrated that TMB testing in ctDNA, measured by hybrid capture-based NGS, correlates to the outcome of NSCLC patients treated with the anti-PD-L1 antibody atezolizumab, with high blood TMB (>16, 394 genes analyzed) being associated with better PFS [96]. In another study, ultra-deep sequencing of serial cfDNA samples obtained from NSCLC patients before and during treatment with the anti-PD-1 antibody pembrolizumab confirmed the association between high baseline TMB level (>21/Mb, 329 genes analyzed) and improved PFS. Moreover, two acquired mutations of *β2-microglobulin* and one of *PTCH1* were identified as possible candidates of resistance [97]. Differently from studies measuring TMB with large panels, in another report a simple algorithm has been developed to predict the outcome of aNSCLC patients treated with PD1 inhibitors, based on cfDNA NGS testing by using a panel of only 36 genes. This panel included variants in genes associated to response (such as *KRAS* and *TP53*) and resistance (such as *STK11* and *PTEN*) to PD1 inhibitors, with the advantage of reducing costs of analysis compared to larger TMB panels [91].

One of the main disadvantages of using blood TMB to predict response to ICIs is that ctDNA is present in low amounts and is indistinguishable from circulating nontumor DNA. Moreover, the presence of alterations of hematopoietic origin in cfDNA, such as *TP53* or *JAK2* mutations, can result in false positive plasma genotyping [98]. Finally, the availability of numerous targeted panels for TMB evaluation and the absence of standardized cut-off values make it difficult to stratify NSCLC patients for treatment with ICIs and further research is still needed in the field of immunotherapy resistance [95].

## 5. Conclusions and Future Perspectives

The above summarized findings clearly indicate that NGS-based testing of cfDNA might have a relevant role in the management of patients with aNSCLC.

Multigene NGS targeted panels should be preferred to standard diagnostic techniques for cfDNA profiling of patients who lack tumor tissue for genotyping, because they allow the identification of many alterations in a single analysis. However, even though NGS methodologies can also reveal the presence of complex genomic variants in cfDNA, such as gene fusions and rearrangements, technology advances are mandatory to improve CNVs detection. Moreover, further studies are required to fully standardize the use of NGS for cfDNA molecular profiling. The presence of DNA nonshedding neoplastic lesions, CHIP or sequencing artifacts affect the sensitivity and specificity of NGS-based cfDNA testing. However, we must acknowledge that most mechanisms responsible for discordance between tumor tissue and cfDNA are independent from the methodology used for profiling.

**Figure 2:**
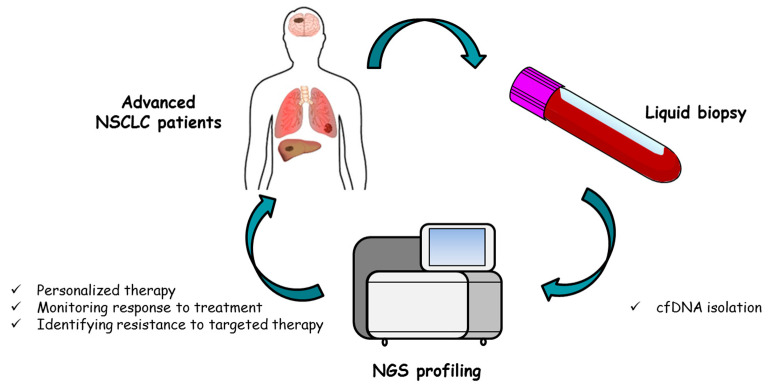
Role of NGS profiling of circulating free DNA in advanced non-small cell lung cancer patients.

The true challenge that we need to face is how to integrate cfDNA testing in the clinical management of lung cancer patients (Figure 2). At present, the use of liquid biopsy is only recommended in cases where tumor tissue is insufficient for genomic profiling. However, liquid biopsy analysis can provide information that is complementary to that of tissue biopsy, especially if NGS techniques are used. In fact, cfDNA analysis allows to better represent tumor heterogeneity which has both a prognostic and a predictive value for molecularly targeted therapies. This information could be of considerable importance for a more appropriate patient stratification, especially when several therapeutic options are available. For example, NSCLC patients carrying *KRAS* mutations will have available as possible treatments in the near future chemotherapy, immunotherapy, chemo-immunotherapy, and targeted therapy. A comprehensive genetic–molecular profile could help to better define which treatment sequence is most appropriate for each patient.

The possibility of tracing multiple mutations in the blood of patients makes cfDNA NGS testing an extremely useful means to evaluate the effectiveness of therapy in the first weeks after its start. This approach may be relevant in patients treated with immunotherapy, to distinguish pseudo-progressions from true progressions, but also in those carrying rare mutations for which the activity of molecularly targeted drugs is not known. Early identification of nonresponders would allow for modification of the therapeutic regimen before the patient’s general condition deteriorates.

Finally, with the introduction in clinical practice of drugs against new molecular targets and of novel agents targeting already established biomarkers, NGS testing of liquid biopsy is indispensable for the identification of mechanisms of acquired resistance and the development of new therapeutic strategies. Although some histological alterations associated with resistance cannot be detected in plasma, cfDNA NGS analysis has the advantage of better representing tumor heterogeneity, which generally increases significantly during the course of the disease due to the development of clones with different mechanisms of resistance in the same tumor site or in distant sites.

An interesting application of cfDNA NGS profiling is the detection of minimal residual disease (MRD) in early-stage or locally advanced NSCLC patients after curative intent therapy. In this regard, NGS-based identification of mutations in liquid biopsies from early stage NSCLC patients after surgical resection or chemotherapy/radiotherapy treatment has demonstrated to predict relapse even months before radiological progression [20,99]. However, large prospective clinical studies are ultimately required to improve MRD detection through cfDNA NGS profiling, with the aim of guiding post-surgical treatment decisions and avoiding unnecessary adjuvant therapies in early stage NSCLC patients.

In conclusion, NGS-based cfDNA testing represents an effective alternative to tissue biopsy in the case of tumor tissue unavailability. Most importantly, it also allows a comprehensive and continuous characterization of the molecular landscape of the whole disease, thus providing a faithful portrait of both spatial and temporal tumor heterogeneity of aNSCLC patients. However, in the challenging era of precision medicine, additional data from prospective clinical trials based on cfDNA NSG testing are needed, in order to implement the translation of research findings into targeted diagnostic procedures and personalized therapies.

## Figures and Tables

**Table 1:** Comparison between next generation sequencing (NGS)-based cfDNA and tumor tissue analyses.

Characteristics of NGS Profiling	cfDNA	Tumor Tissue
Minimally invasive procedure	√	-
Easy repeatability	√	-
Standardized methodologies	-	√
Short turnaround times	√	-
High concentration of tumor DNA	-	√
Low contamination from nontumor DNA	-	√
Comprehensive tumor profile	√	√
Tumor heterogeneity	Intratumor and interlesions	Intratumor only
Real-time monitoring of disease	√	-
Early detection of resistance	√	-

**Table 2:** Characteristics of the main NGS methodologies used for liquid biopsy analysis.

NGS Methodology	LoD (%)	Advantages	Pitfalls
Whole genome sequencing and whole exome sequencing	_	Identification of new targets and mechanisms of resistance	Long turnaround times, heavy bioinformatics, high costs
Amplicon-based targeted sequencing			
TAm-Seq	2%	Flexibility, cost-effectiveness	Identification of only SNVs, high detection limit
eTAm-Seq	0.25%	Identification of SNVs, CNVs and short indels	Sequencing of a limited number of predefined hotspot mutations
Safe-SeqS	0.01%	Reduced error rate of 70%	Requirement of a gel-purification step
AmpliSeq HD	0.1%	Low detection limit, low error rate	Sequencing of a limited number of predefined hotspot variants
Capture-based targeted sequencing			
CAPP-Seq	0.02%	Sequencing of a high number of genes	High error rate
iDES	0.0025%	Very low detection limit	High amount of DNA input required

Legend: LoD, limit of detection; TAm-Seq, Tagged-Amplicon deep Sequencing; SNVs, single nucleotide variations; eTAm-Seq, enhanced TAm-Seq; CNVs, copy number variations; Safe-SeqS, Safe-Sequencing System; CAPP-Seq, CAncer Personalized Profiling by deep Sequencing; iDES, integrated Digital Error Suppression.

**Table 3:** Increase of NSCLC patients with at least one biomarker identified using cfDNA NGS analysis in addition to tumor tissue testing.

Author	N° of Patients	Tumor Tissue Biomarker Positive (%)	Tumor Tissue and cfDNA Biomarker Positive (%)	Increase of Biomarker Detection (%)
Mack et al. [43]	1288	383 (29.7)	635 (49.3)	65%
Leighl et al. [44]	282	60 (21.3)	89 (31.5)	48%
Aggarwal et al. [24]	229	47 (20.5)	82 (35.8)	75%
